# Proteasome Activators, PA28*α* and PA28*β*, Govern Development of Microvascular Injury in Diabetic Nephropathy and Retinopathy

**DOI:** 10.1155/2016/3846573

**Published:** 2016-10-18

**Authors:** Saeed Yadranji Aghdam, Ali Mahmoudpour

**Affiliations:** ^1^Reynolds Institute on Aging, Room No. 4151, 629 Jack Stephens Drive, Little Rock, AR 72205, USA; ^2^Department of Geriatrics, University of Arkansas for Medical Sciences, Little Rock, AR, USA; ^3^Norgen Biotek Corp., 3430 Schmon Parkway, Thorold, ON, Canada L2V 4Y6

## Abstract

Diabetic nephropathy (DN) and diabetic retinopathy (DR) are major complications of type 1 and type 2 diabetes. DN and DR are mainly caused by injury to the perivascular supporting cells, the mesangial cells within the glomerulus, and the pericytes in the retina. The genes and molecular mechanisms predisposing retinal and glomerular pericytes to diabetic injury are poorly characterized. In this study, the genetic deletion of proteasome activator genes, PA28*α* and PA28*β* genes, protected the diabetic mice in the experimental STZ-induced diabetes model against renal injury and retinal microvascular injury and prolonged their survival compared with wild type STZ diabetic mice. The improved wellbeing and reduced renal damage was associated with diminished expression of Osteopontin (OPN) and Monocyte Chemoattractant Protein-1 (MCP-1) in the glomeruli of STZ-injected PA28*α*/PA28*β* double knockout (Pa28*αβ*DKO) mice and also in cultured mesangial cells and retinal pericytes isolated from Pa28*αβ*DKO mice that were grown in high glucose. The mesangial PA28-mediated expression of OPN under high glucose conditions was suppressed by peptides capable of inhibiting the binding of PA28 to the 20S proteasome. Collectively, our findings demonstrate that diabetic hyperglycemia promotes PA28-mediated alteration of proteasome activity in vulnerable perivascular cells resulting in microvascular injury and development of DN and DR.

## 1. Introduction

Diabetic high blood glucose or hyperglycemia causes mortality and morbidity through DN and DR via disrupting the vascular function in the kidney and retina. These pathologies are the major cause of death associated with renal failure and blindness among the type 1 and type 2 diabetes patients [1–3]. Similar molecular pathways appear to govern the development of diabetic renal and retinal microvascular injury. This speculation arises from higher coincidence rates of DN and DR; that is, patients with DN have already developed DR and patients with DR are vulnerable to develop DN [4, 5]. According to the similarities in pathologic background affecting the retina and kidney, it was hypothesized that DN and DR could arise by injuries to perivascular supporting cells in glomeruli and retina. The vulnerable vascular cell types affected by diabetic hyperglycemia are retinal pericytes (RPC) and their analogs within glomeruli are the mesangial cells. In DR, the RPC undergo cell death and disengage from the retinal vasculature, predisposing the retina to neoangiogenesis, vascular leakage, and tractional retinal detachment culminating in reduced vision or terminal blindness [6–8]. DN is characterized by mesangial matrix expansion and the obstruction of the glomerular capillaries within the renal filtration units, the glomeruli [9]. The glomerulopathy in DN is associated with reduced efficiency of renal filtration, and in chronically established cases it triggers end-stage renal failure and mortality [10].

Several biochemical mechanisms and pathways have been described in speculation of the pathogenesis caused by either DN or DR. These mechanisms include enhanced oxidative stress, enhanced polyol pathway, PKC activation, inflammation, and advanced glycation end product formation [3, 11–13]. The most extensively investigated mechanisms associated with the development of both DN and DR are enhanced oxidative stress and inflammation caused by metabolic alterations. However, a plethora of evidence indicates that immunological and inflammatory mechanisms are important factors in development and progression of DR and DN [14–17]. The recruitment of the activated macrophages and increased generation of inflammatory and proinflammatory mediators (TNF-*α*, IL-1*β*, IL-6, MCP-1, and OPN) in retinal and renal milieu is linked to the progression and exacerbation of the DN and DR [17–22]. However, the exact molecular and cellular mechanisms involved have remained elusive.

Oxidative stress is a phenomenon in which the balance between the generation of free oxidizing radicals and the system responsible for their removal in the cell is disturbed leading to enhanced formation of free reactive ions including reactive oxygen species (ROS). The increased ROS levels in cells subjected to diabetic hyperglycemia drive the antioxidant gene expression to protect the cells from oxidative injuries [23]. One of the target genes to be induced following oxidative stress is the NF-E2-related transcription factor 2 (Nrf2). Two of the recently characterized genes that are regulated by the Nrf-2 and provide protection against oxidative stress and stress-adaptation are proteasome activator genes, PA28*α* and PA28*β* [24]. It is speculated that PA28 proteins increase the proteasomal degradation activity to clear the oxidized or misfolded proteins [24–26]. The PA28*α*/*β* genes were initially identified as components of immunoproteasomes which are induced in response to interferon-*γ*. PA28*α* and PA28*β* proteins form a heptameric complex (4*β*/3*α*) acting as the gate opener for the 20S proteasomes, hence stimulating the degradation of the nonubiquitinated short peptides. The well-characterized function for the PA28 proteins is the generation of the antigenic peptides to be presented by the MHC class I molecules [27, 28].

The PA28*α*/*β* are upregulated in the cultured RPC under high glucose conditions and also in the intraglomerular capillaries of older type 1 diabetic Akita mice [1]. To understand the role of PA28*α*/*β* genes in development of DN and DR, Pa28*αβ*DKO mice were tested and their physiological and biometric indices were compared with STZ-induced diabetic wild type mice. The diabetic Pa28*αβ*DKO mice provided higher survival rate, higher body weight, more efficient renal filtration function, and reduced microvascular damage in their retinae compared to diabetic wild type mice. The glomeruli, mesangial cells, and the RPC isolated from Pa28*αβ*DKO mice had lower levels of OPN and MCP-1 under high glucose conditions. OPN is known as a proinflammatory protein associated with progression of diabetic microvascular injury [19, 22]. The PA28-dependent regulation of OPN expression was stimulated by high glucose and abrogated by synthetic peptides that block the binding of PA28 to 20S proteasomes. Therefore, the findings of this study provide novel insights into the role of the ubiquitin proteasome system (UPS) and specially the PA28 proteins, in regulating the microvascular injury in diabetes.

## 2. Material and Methods

### 2.1. Animals

Animal maintenance, genotyping, treatments, and analytical procedures followed the guidelines accredited by Institutional Animal Care and Use Committee of the University of Wisconsin, School of Medicine and Public Health. All the mice used for the experiments were in C57BL/6 background. For cell isolation, 6-week old male immorto mice (stock number 006553) and to study diabetes, Pa28*αβ*DKO male mice (stock number 021202) were used. Diabetes was induced in animals with a single injection of streptozotocin (STZ; 180 mg/kg in citrate buffer, pH 4.2 by ip injection). Three days following the treatment, the blood glucose of all STZ-injected animals was above 400 mg/dL.

### 2.2. Albumin-to-Creatinine Ratio (ACR) Analysis

For ACR analysis the urine samples were collected by housing each individual mouse in a metabolic cage (Tecniplast, Italy) for 24 hours. Urinary albumin and creatinine levels were measured by ELISA (Albuwell M, Exocell) and the ACR measurements were conducted according to the guidelines provided by the manufacturer.

### 2.3. Transmission Electron Microscopy (TEM) and Histological Analysis

Mouse kidneys were sliced and immersion-fixed in a solution of 2% paraformaldehyde (PFA) and 2.5% glutaraldehyde in 0.1 M sodium cacodylate buffer, pH 7.4, overnight at 4°C. The tissue was postfixed at room temperature for 2 hours in 1% osmium tetroxide in the same buffer. Subsequently the samples were dehydrated in a graded ethanol series, then dehydrated in propylene oxide, and embedded in Epon epoxy resin. Ultrathin sections were prepared using Leica UC6 Ultramicrotome and mounted on 200-mesh carbon-coated copper grids. Tissue sections were observed with a Philips CM120 electron microscope, and images were captured with a MegaView III side-mounted digital camera.

For PAS and JMS-H&E staining the formalin fixed, paraffin embedded, 5-6 *μ*m tissue sections were deparaffinized in xylene and rehydrated in descending graded percentage of ethanol (100%, 95%, 80%, and 70%) and routinely stained with recommended reagents. Digital images were taken with NA PL APO objectives (10x/0.25 NA, 40x/0.95 NA, and 63x/1.4 NA oil) on a ScanScope XT system using ImageScope version 10 software (Aperio Technologies Inc.).

### 2.4. Cell Isolation, Culture, and Peptide Transfections

The isolation and culture of the RPC was described before [29]. Briefly, the retinae from one litter (6-7 pups, 6-week old) immorto mice were collected under a dissecting microscope. The collected retinae were rinsed with serum-free Dulbecco's Modified Eagle's Medium (DMEM), pooled, minced, and digested for 45 min with collagenase type II (1 mg/mL, Worthington) with 0.1% BSA in serum-free DMEM at 37°C. Cells were resuspended in equal amount of DMEM containing 10% Fetal Bovine Serum (FBS) and spun for 5 min at 400 ×g. The pelleted cells were resuspended in 4 mL DMEM containing 10% FBS, 2 mM L-glutamine, 100 *μ*g/mL streptomycin, 100 U/mL penicillin, and recombinant murine IFN-*γ* (R&D Systems) at 44 U/mL. Cells were evenly divided into 4 wells of a 24-well tissue culture plate and maintained at 33°C with 5% CO_2_. Cells were progressively passed to larger plates and maintained and propagated in 60 mm dishes.

The isolation and culture of mouse REC was described elsewhere [30]. The retinae from one litter (6 to 7 pups, 6-week old) of immorto mice were dissected out aseptically under a dissecting microscope and kept in HBSS buffer containing penicillin/streptomycin. Retinae were pooled together, rinsed with HBSS buffer (Life Technologies), minced into small pieces in a 60 mm tissue culture dish using sterile razor blades, and digested in 5 mL of collagenase type I (1 mg/mL in serum-free DMEM, Worthington) for 45 min at 37°C. Following digestion, DMEM with 10% FBS was added and cells were pelleted. The cells were passed through a sterile 40 *μ*m nylon strainer (BD Falcon) and spun at 400 ×g for 10 min to pellet cells, and cells were washed twice with DMEM containing 10% FBS. The cells were resuspended in 1.5 mL medium (DMEM with 10% FBS) and incubated with anti-PECAM-1 antibody-conjugated Dynabeads (Life Technologies). After affinity binding, magnetic beads were rinsed six times with DMEM with 10% FBS and bound cells in endothelial cell growth medium were plated into a single well of a 24-well plate precoated with 2 *μ*g/mL of human fibronectin (BD Biosciences). Endothelial cells were grown in DMEM containing 20% FBS, 2 mM L-glutamine, 2 mM sodium pyruvate, 20 mM HEPES, 1% nonessential amino acids, 100 *μ*g/mL streptomycin, 100 U/mL penicillin, freshly added heparin at 55 U/mL (Sigma), endothelial growth supplement 100 *μ*g/mL (Sigma), and recombinant murine IFN-*γ* (R&D Systems) at 44 U/mL. Cells were maintained at 33°C with 5% CO_2_. Cells were progressively passed to larger plates, maintained, and propagated in 1% gelatin-coated 60 mm dishes.

The glomeruli and mesangial cells were isolated using a standard procedure described before [31]. Briefly, 8 × 10^7^ Dynabeads (Life Technologies) were diluted in 40 mL of phosphate-buffered saline and perfused through the heart of the adult mice. The kidneys were extracted, mechanically minced, digested with type 1 collagenase, and filtered through 100 *μ*m strainer. The glomeruli were collected with a magnet and washed three times to eliminate the nonglomerular cells and debris. The glomeruli were grown in either pericytes growth medium to establish the mesangial cell culture.

The KGEC were isolated from the glomeruli similar to mesangial cell isolation following a procedure described before [32]. The purified glomeruli were seeded and allowed to grow in REC medium on coated 35 mm plates. Upon 80–85% confluency, the cells were incubated with anti-PECAM-1 antibody-conjugated Dynabeads, washed, and enriched similar to REC cultures. The purity of the isolated cells was determined by FACS and immunostaining. The BPC, BEC, KPC, HPC, and LPC were isolated by following a procedure similar to RPC isolation. The peptides (CPC Scientific) and the Chariot transfection reagent (Active Motif) were reconstituted and used according to the manufacturer guidelines.

### 2.5. Trypsin-Digested Retinal Vascular Preparations

The enucleated eyes were fixed in 4% paraformaldehyde for at least 24 h. The dissected retinae were washed overnight in deionized water and incubated in 3% Trypsin (Difco™ Trypsin 250, Becton Dickinson and Company) prepared in 0.1 M Tris, 0.1 M maleic acid, and pH 7.8 containing 0.2 M NaF for 2 h at 37°C. The neuroretinal tissue was gently brushed away, and the resultant isolated vascular tree was air dried onto a glass microscope slide [33].

### 2.6. RNA Isolation and Real-Time PCR

Total RNA from cells was extracted by total RNA isolation kit (Norgenbiotek) according to the manufacturer's instructions. Cells were allowed to reach 85–90% confluence, rinsed twice with PBS, scraped off the plates, and transferred to RNase-free microfuge tubes. The RNA was purified using Trizol reagent (Life Technologies). The cDNA synthesis was performed with 1 *μ*g of total RNA. One microliter of 1/10-diluted cDNA was used as real-time PCR template in triplicate groups and reaction was completed using SYBR Green master mix in Mastercycler Realplex (Eppendorf) with the specified primers in Supplemental Experimental Procedures. Thermal cycles were programmed as 95°C for 2 min; 40 cycles of amplification (95°C for 15 s and 60°C for 40 s); and a dissociation curve step (95°C for 15 s, 60°C for 15 s, and 95°C for 15 s). Standard curves were generated from known quantities for each target gene of linearized plasmid DNA. Tenfold diluted series were used for each known target to be amplified by SYBR Green qPCR premix. The linear regression line for ng of DNA was calculated from relative fluorescent units at a threshold fluorescence value (Ct) to quantify amplification targets from cell extracts by comparing the relative fluorescent units at the Ct to the standard curve, normalized by the simultaneous amplification of RpL13A (a housekeeping gene) for all samples.

### 2.7. Protein Isolation and Western Blot

For protein isolation, the cells in 80% confluent dishes were harvested following adding the lysis buffer (150 mM NaCl, 1% Triton X-100, 20 mM Tris-HCl (pH 7.4), protease inhibitor cocktail (1 mM PMSF, 10 mM Leupeptin, 10 mM Pepstatin)). For western blot analysis, 50 *μ*g cell lysate or conditioned medium proteins were mixed with Laemmli sample buffer and the reduced or nonreduced samples were boiled for 10 min. The boiled samples were loaded into 4–20% Tris Glycine acrylamide gels (Life Technologies) and electrophoresis was performed using SDS running buffer (25 mM Tris, 190 mM glycine, 0.1% SDS, pH 8.3).

### 2.8. Plasmids and Cell Transfections

Mouse cDNA encoding the PA28*α* and PA28*β* were cloned into pLVX-IRES-Hyg and pLVX-IRES-Neo plasmids (Clontech), respectively. The lentiviral particles were prepared by transfecting the Lenti-X 293T cells (Clontech) and collecting the conditioned media. The pBABE-OPN/Puro plasmid and the packaging cells are described elsewhere [34]. Cultured cells were transfected using the FuGENE6 reagent (Promega) according to recommended protocol. After 48 h, Hygromycin B (500 *μ*g/mL; Sigma-Aldrich), Neomycin (750 *μ*g/mL; Life Technologies), and Puromycin (5 *μ*g/mL; Sigma-Aldrich) were added to cell cultures.

### 2.9. Statistical Analysis

The data are represented as the mean ± SEM. Lifespan analyses were performed using Kaplan-Meier survival analysis. Statistical significance was determined by analysis of variance (ANOVA) and Tukey-Kramer post hoc analysis for multiple comparisons using *p* value of 0.05 in GraphPad Prism software (San Diego, CA, USA). *p* values *p* < 0.05 were considered significant.

## 3. Results and Discussion

### 3.1. PA28*α* and PA28*β* Regulate the Survival Rate and Renal Filtration Efficiency in STZ-Induced Diabetic Mice

The PA28 proteasome regulators were previously shown to be dramatically upregulated in the glomerular capillaries of Akita mouse model with chronic hyperglycemia for 8 months but not in the same age wild type nondiabetic mice [1]. In this study the role of PA28*α*/*β* in modulating diabetic microvascular disorders was investigated via streptozotocin (STZ) injection to both wild type and Pa28*αβ*DKO mice. STZ has cytotoxic effects on insulin-secreting pancreatic *β*-cells and induces type I diabetes in experimental animals [35].

**Figure 1:**
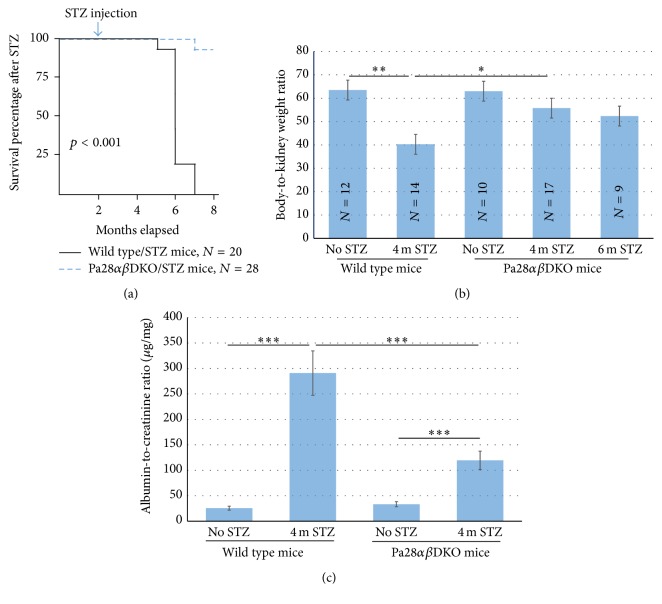
Pa28*αβ*DKO/STZ mice provide higher survival rate and better renal filtration efficiency than wild type/STZ mice. (a) Survival rate of wild type and Pa28*αβ*DKO mice following STZ injection. *N* represents the number of animals in each experimental group. (b) Body-to-kidney weight ratio in wild type and Pa28*αβ*DKO mice before and after STZ injection for four and six months. (c) Albumin-to-creatinine ratio in urine samples of wild type and Pa28*αβ*DKO mice before and after four months of STZ injection. Results presented as mean ± SEM. ^*∗*^ *p* < 0.05, ^*∗∗*^ *p* < 0.01, and ^*∗∗∗*^ *p* < 0.001.

All the Pa28*αβ*DKO/STZ and wild type/STZ mice had blood glucose levels above 450 mg/dL three days after STZ injection and on the day of sacrifice (Table 1). In comparison with Pa28*αβ*DKO/STZ mice, the wild type/STZ mice exhibited significantly reduced survival rates and weight loss by four months of STZ injection. The Pa28*αβ*DKO/STZ mice were protected and had no obvious weight loss or reduced survival after six months of STZ injection (Figures 1(a) and 1(b)). It was assumed that improved renal function was responsible for the observed improvement in the health of the Pa28*αβ*DKO/STZ mice. To test this, the albumin-to-creatinine ratio (ACR) test was used. ACR is a cornerstone assay for the diagnosis of renal filtration disorders [36, 37] and reflects the efficiency of the kidney filtration function. The measured albuminuria was significantly higher in wild type/STZ (291 ± 8.54 *μ*g/mg) compared to that of non-STZ wild type (25.61 ± 2.18 *μ*g/mg) mice and 28*αβ*DKO/STZ mice (119.6 ± 5.61 *μ*g/mg) (Figure 1(c)).

**Figure 2:**
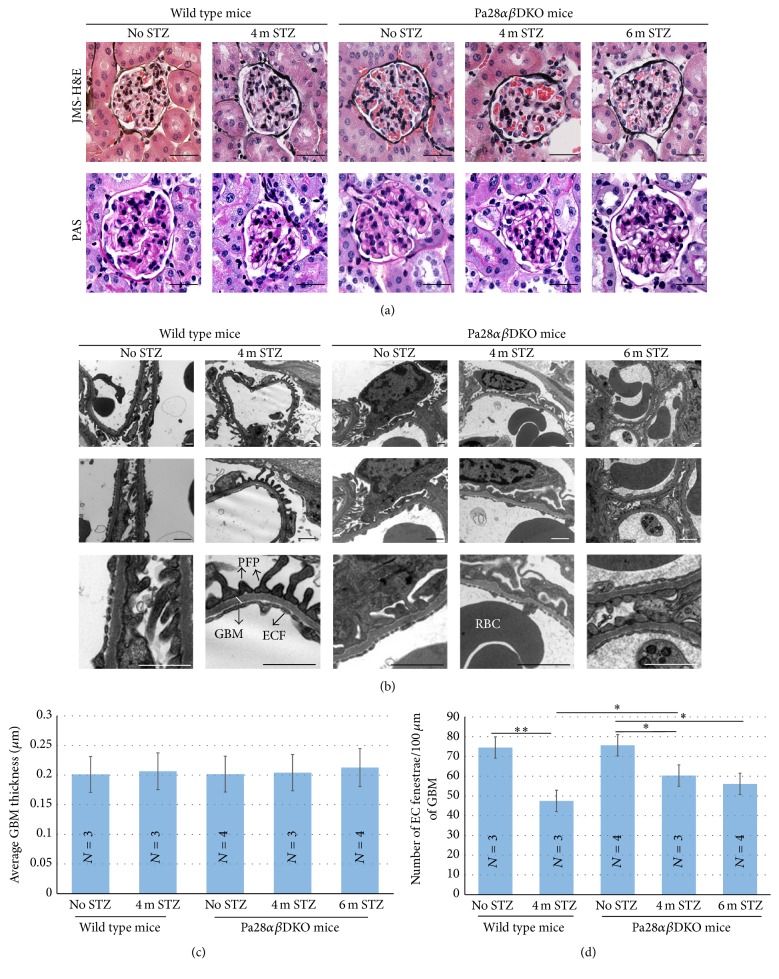
Light microscopic and TEM analysis of the glomeruli. (a) JMS-H&E and PAS staining of the nondiabetic and STZ-diabetic wild type and Pa28*αβ*DKO glomeruli. Scale bars, 30 *μ*m. (b) Ultrastructure of the glomeruli in wild type and Pa28*αβ*DKO mice before and after STZ injection for indicated time points. Glomerular basement membrane (GBM), podocyte foot processes (PFP), endothelial cell fenestrae (ECF), and red blood cell (RBC) are shown. Scale bars, 1 *μ*m. (c) Quantitation of the GBM thickness in TEM images of wild type and Pa28*αβ*DKO glomeruli. (d) Quantitation of the average number of the EC fenestrae in TEM images of wild type and Pa28*αβ*DKO glomeruli. N represents the number of animals in each experimental group. Results presented as mean ± SEM. ^*∗*^ *p* < 0.05, ^*∗∗*^ *p* < 0.01.

Next the histological and ultrastructural properties of the glomeruli in the non-STZ and STZ-injected mice were assessed. Renal histology in kidney sections was evaluated using the combined Jones Methenamine Silver- (JMS-) Hematoxylin and Eosin (H&E) staining and also Periodic Acid Schiff (PAS) staining. The JMS and PAS staining is routinely used to detect the mesangial matrix increase and changes in the glomerular basement membrane (GBM) [38]. There were no discernible alterations in the appearance of GBMs, the adjacent tubules, and also the mesangial or endothelial cells in different animal groups (Figure 2(a)).

**Figure 3:**
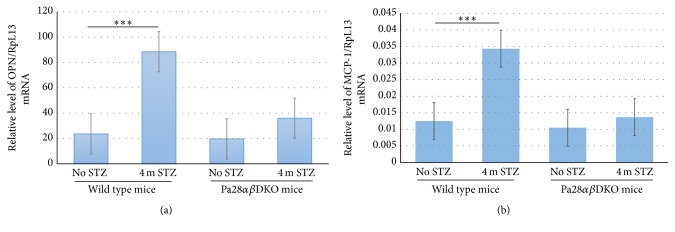
Deletion of the PA28*α* and PA28*β* genes suppresses the glomerular expression of OPN and MCP-1 in STZ-injected mice. (a) qPCR analysis of OPN and (b) MCP-1 mRNA in wild type and Pa28*αβ*DKO glomeruli without or with four months of STZ injection. *n* = 3 per group. Results presented as mean ± SEM. ^*∗∗∗*^ *p* < 0.001.

Similar to light microscopic analysis, TEM analysis did not show any significant difference in the thickness of GBM in wild type/STZ and Pa28*αβ*DKO/STZ mice. The lack of visible diabetic glomerular pathology in TEM test despite the reduced renal filtration efficiency (determined by ACR test) in mice studied here is likely due to the resistance of the C57BL/6 mouse line against morphological changes in GBM (Figures 2(b) and 2(c)). However, there was a significant reduction in the relative number of intraglomerular endothelial fenestrae in four-month STZ-injected wild type mice compared to those of Pa28*αβ*DKO/STZ mice (47.5 ± 1.74 versus 60.3 ± 0.67) (Figure 2(d)). Similarly, the six-month STZ-injected Pa28*αβ*DKO mice provided higher endothelial fenestrae compared with four-month STZ-injected wild type mice (56 ± 0.93 versus 47.5 ± 1.74) (Figure 2(d)). Since the inflammatory mediators OPN and MCP-1 have been shown to be closely associated with the development and progression of the DN and DR, we investigated their levels using real-time PCR in the glomerular mRNA isolated from STZ-injected and nondiabetic wild type and Pa28*αβ*DKO mice. The qPCR analyses did not provide any differences in the levels of IL-1*β* and TNF-*α* mRNA among wild type and Pa28*αβ*DKO mice under both normal and STZ- injected conditions (not shown). However, the mRNA for OPN and MCP-1 showed significant increase in wild type/STZ versus nondiabetic wild type glomeruli (Figures 3(a) and 3(b)). Interestingly, unlike the wild type/STZ glomeruli, in Pa28*αβ*DKO/STZ glomeruli the expression of OPN and MCP-1 was not altered significantly. These data demonstrate that PA28*α* and PA28*β* regulate the diabetic glomerular vascular injuries most likely through regulating the expression of proinflammatory agents.

### 3.2. Cultured Pa28*αβ*DKO Mesangial Cells Express Reduced Quantities of OPN and MCP-1 under High Glucose Conditions

**Figure 4:**
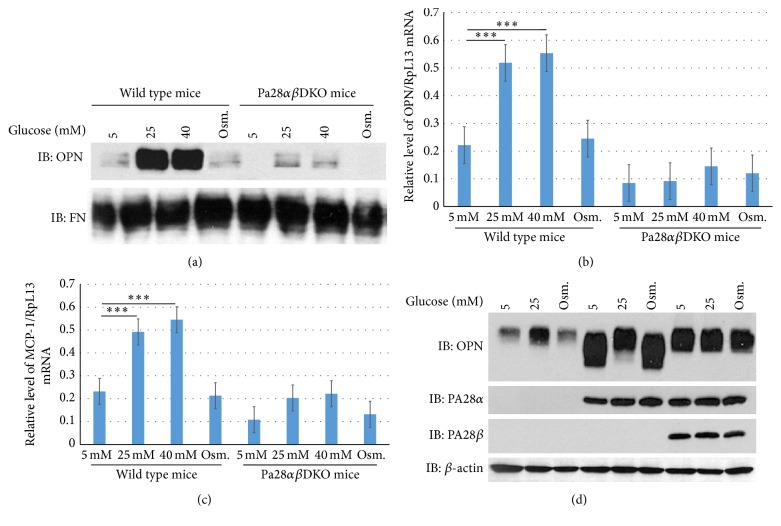
PA28*α* and PA28*β* regulate the expression of OPN in cultured mesangial cells under high glucose concentrations. (a) Western blot analysis of the conditioned media of cultured wild type and Pa28*αβ*DKO mesangial cells under different glucose conditions and osmolarity treatment. OPN: Osteopontin and FN: Fibronectin. (b) qPCR analysis of OPN and (c) MCP-1 mRNA in wild type and Pa28*αβ*DKO mesangial cells under different glucose concentrations. (d) Western blot analysis for OPN in the conditioned media and PA28*α*, PA28*β*, and *β*-actin in the cell lysate following lentiviral reexpression of PA28*α* and PA28*α*/PA28*β* in Pa28*αβ*DKO mesangial cells. Results presented as mean ± SEM. ^*∗∗∗*^ *p* < 0.001.

To understand how PA28*α* and PA28*β* genes promote glomerular injuries during diabetes, as an in vitro model, the mesangial cells from wild type and Pa28*αβ*DKO mice were isolated for further characterizations [29, 31, 39]. Since the mRNA levels for OPN and MCP-1 in Pa28*αβ*DKO/STZ glomeruli were lower than those of wild type/STZ mice and the expression of these proteins has been linked to the development of DN and DR, we investigated whether higher glucose concentrations in vitro could induce the expression of OPN and MCP-1 genes in isolated mesangial cells. Western blot and qPCR analysis of these genes were performed following three days of treatment with either normal (5 mM D-glucose), high glucose (25 mM or 40 mM D-glucose), or osmolarity control (5 mM D-glucose plus 20 mM L-glucose). Western blot analysis of the conditioned medium provided robust induction of OPN expression in wild type but not in Pa28*αβ*DKO mesangial cells following treatment with high glucose (Figure 4(a)). The qPCR analysis of wild type and Pa28*αβ*DKO cells also confirmed the upregulation of OPN expression in wild type but not in Pa28*αβ*DKO mesangial cells treated with high glucose (Figure 4(b)). Because MCP-1 protein could not be detected by western blot (not shown), we used qPCR to evaluate changes in MCP-1 transcript levels in cultured mesangial cells under normal and high glucose conditions. Consistent with the qPCR data for OPN, the MCP-1 transcripts were significantly increased in the wild type mesangial cells grown under high glucose conditions, whereas the Pa28*αβ*DKO cells had markedly lower levels of MCP-1 transcripts at the same conditions (Figure 4(c)).

It was next tested whether lentiviral-mediated reexpression of the PA28*α* and PA28*β* individually or simultaneously restored the OPN expression in Pa28*αβ*DKO mesangial cells. The reexpression of PA28*α* alone or PA28*α* and PA28*β* together restored the OPN release from Pa28*αβ*DKO cells under both normal and high glucose conditions (Figure 4(d)). The overexpression of PA28*α* resulted in less vigorous expression of OPN compared to simultaneous overexpression of both PA28*α* and PA28*β*. This effect could be attributed to the ability of the PA28*α* subunits to form an active homoheptameric proteasome regulatory complex that has not been reported for PA28*β* monomers [28].

### 3.3. Pa28*αβ*DKO/STZ Mice Provide Lower Number of Retinal Acellular Capillaries

**Figure 5:**
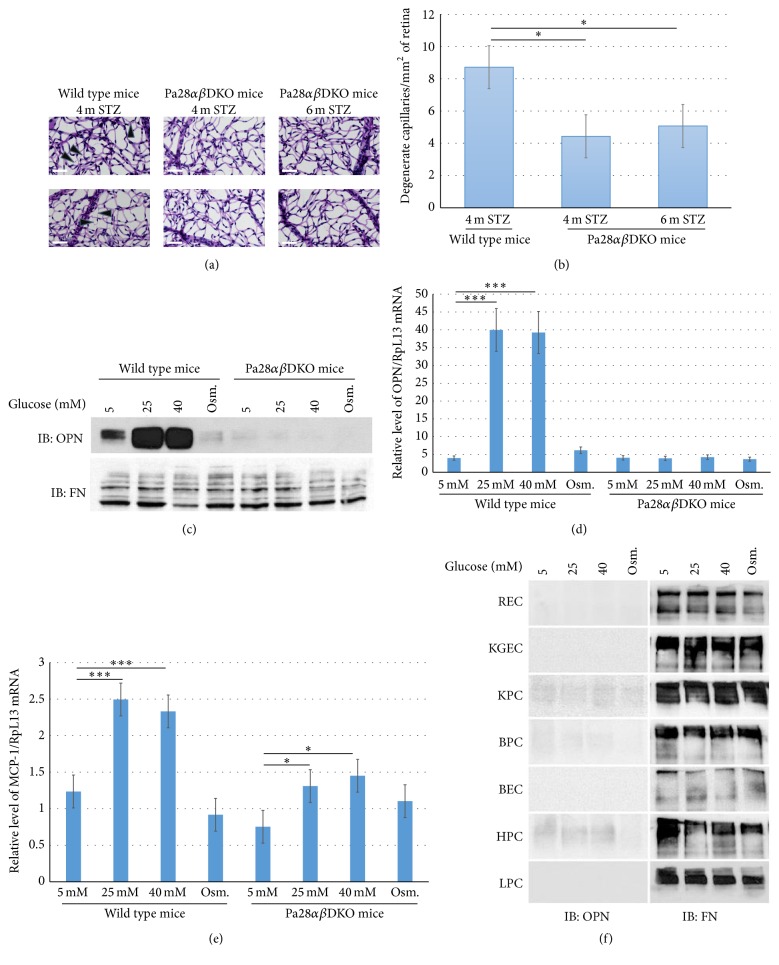
PA28*α* and PA28*β* regulate the diabetic retinal microvascular injury. (a) Light microscopic images of the flat mounted retinae from wild type and Pa28*αβ*DKO mice following four or six months of STZ injection. The arrowheads point to the degenerate capillaries. Scale bars, 10 *μ*m. (b) Quantitation of the number of degenerate capillaries in wild type/STZ and Pa28*αβ*DKO/SZT mice. (c) Western blot analysis of the conditioned media of cultured wild type and Pa28*αβ*DKO RPC under different glucose conditions and osmolarity treatment. (d) qPCR analysis of OPN and (e) MCP-1 mRNA in wild type and Pa28*αβ*DKO RPC under different glucose concentrations. (f) Western blot analysis of OPN and FN in the conditioned media obtained from various vascular and perivascular cell types under different glucose conditions. Results presented as mean ± SEM. ^*∗*^ *p* < 0.05, ^*∗∗∗*^ *p* < 0.001.

DR is another microvascular complication of diabetes attributed to the diminution of pericytes in retinal microvessels and subsequent death of endothelial cells. In retina, such obsolete blood vessels will appear as thinner entities known as acellular capillaries [40]. Therefore, we investigated to test in respect of protection against glomerular microvascular injury in Pa28*αβ*DKO/STZ mice whether these mice were similarly protected against microvascular injury in the retina. Compared with four-month STZ-injected wild type mice, the prepared retinal flat mounted microvessels of Pa28*αβ*DKO mice showed remarkable reduction in the number of acellular capillaries after four months (8.71 ± 0.43 versus 4.43 ± 0.45) and six months of STZ injection (8.71 ± 0.43 versus 5.07 ± 0.28), respectively (Figures 5(a) and 5(b)).

Reduction in the number of the acellular capillaries in Pa28*αβ*DKO/STZ retinae demonstrated that loss of PA28*α*/*β* genes protected the RPC against cell death triggered by hyperglycemia. To assay the effect of high glucose treatment on cultured RPC and investigate whether they respond to high glucose the same as to mesangial cells, RPC were isolated and cultured from wild type and Pa28*αβ*DKO mice. High glucose treatment of wild type RPC robustly induced the expression of OPN and MCP-1 while the Pa28*αβ*DKO RPC treated with high glucose did not manifest such increase in OPN and MCP-1 expression (Figures 5(c)–5(e)). Next it was tested whether increased expression of OPN in response to high glucose was uniquely restricted to RPC and mesangial cells or other vascular cell types also responded similarly. For this purpose, different mouse endothelial and perivascular cell types were isolated from various organs and exposed to high glucose similar to RPC and mesangial cells. These cells included retinal endothelial cells (REC), kidney glomerular endothelial cells (KGEC), total kidney nonglomerular pericytes (KPC), brain pericytes (BPC), brain endothelial cells (BEC), heart pericytes (HPC), and lung pericytes (LPC). Western blot analyses of OPN demonstrated that any of these vascular cell types did not contain elevated OPN production under high glucose conditions (Figure 5(f)). Hence, increased production of OPN under high glucose conditions is a unique characteristic of RPC and mesangial cells.

### 3.4. The XAPC7 and HBX Peptides Inhibit the OPN Release from Cultured Mesangial Cells under High Glucose Conditions

**Figure 6:**
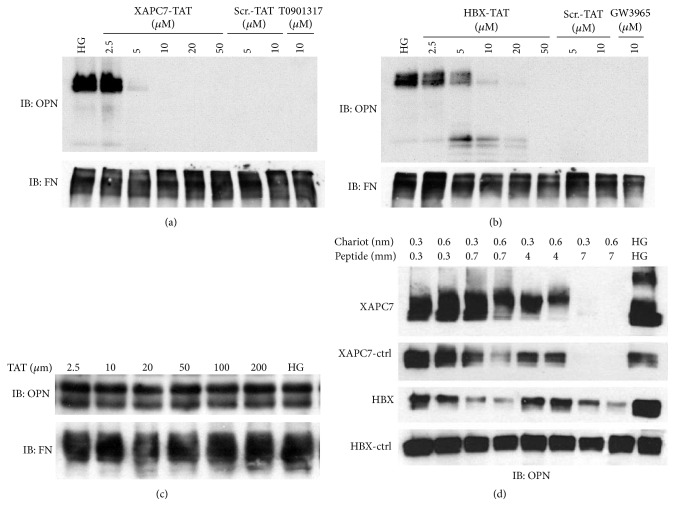
The XAPC7 and HBX peptides inhibit the high glucose-induced OPN production in cultured mesangial cells. (a) Western blot analysis of the conditioned media of cultured wild type mesangial cells treated with XAPC7-TAT and scrambled XAPC7-TAT or (b) HBX-TAT and scrambled HBX-TAT peptides under high glucose (HG, 25 mM) conditions. (c) Western blot analysis of the conditioned media of cultured wild type mesangial cells treated with TAT peptide under high glucose (25 mM) conditions. (d) Western blot analysis of the conditioned media of cultured wild type mesangial cells treated with Chariot/XAPC7, Chariot/HBX, and Chariot/scramble peptides under high glucose conditions (25 mM).

To investigate if the OPN release from mesangial cells exposed to high glucose was caused by the binding of Pa28*α*/*β* proteins to the 20S proteasomes, we examined peptides capable of disrupting the association of Pa28*α*/*β* with the 20S proteasome. The PA28-inhibitory peptides fused to TAT domain of the HIV virus were used for this assay [41]. TAT sequence was added to the XAPC7 and HBX peptides to promote the transduction of the cultured cells. The XAPC7-TAT and HBX-TAT peptides as well as scrambled control peptides fused to TAT were used to transfect the cultured mesangial cells for three days under high glucose condition to promote OPN release. Western blot analyses of conditioned media provided strong inhibition of OPN release after treatment with XAPC7-TAT and HBX-TAT at 5 *μ*M and 10 *μ*M concentrations, respectively (Figures 6(a) and 6(b)). Treatment with the liver X receptor (LXR) agonists, T0901317 and GW3965, was shown to inhibit OPN release and ameliorate the severity of DN [42]. Hence, as positive control for our peptide treatment experiments and suppression of OPN release, we used LXR agonists in cotreatment of the mesangial cells with high glucose. As a result, almost both T0901317 and GW3965 completely inhibited the OPN release at 10 *μ*M concentrations (Figures 6(a) and 6(b)). Interestingly, the TAT-fused scrambled peptides also suppressed OPN release in response to high glucose. TAT peptide had been previously demonstrated to inhibit the PA28 binding to the 20S proteasomes [43, 44]. Therefore, the inhibitory effect of the TAT-scrambled peptides could be attributed to the TAT domain of the peptides. However, treatment of the mesangial cells with TAT-only peptide even at 200 *μ*M concentration did not inhibit the OPN release (Figure 6(c)). The failure of the TAT-only peptide in inhibiting the OPN release under high glucose conditions suggests that TAT requires fusion to a juxtaposing peptide to inhibit PA28 binding to the 20S proteasome. In order to exclude the inhibitory effect of the TAT domain, XAPC7 and HBX peptides without TAT domain were synthesized and delivered to the mesangial cells using Chariot transfection reagent. Treatment with XAPC7 and its scrambled control resulted in complete dose-dependent inhibition of the OPN release (Figure 6(d)). This effect of control peptide is possibly caused by the shorter size and positive charge of these peptides compared with HBX peptide. The HBX showed marked reduction in OPN release at various concentrations but this inhibition was less potent than XAPC7. The scrambled HBX peptide did not markedly affect the OPN release from the mesangial cells (Figure 6(d)). Collectively, these findings demonstrate that high glucose-induced binding of Pa28*α* and Pa28*β* to 20S proteasomes in RPC and mesangial cells regulates OPN production.

Taking advantage of the Pa28*αβ*DKO mice, the mesangial cells, and RPC isolated from Pa28*αβ*DKO mice it was demonstrated in this study that PA28*α* and PA28*β* genes are key determinants of diabetic microvascular injuries. Evidence is provided for the involvement of PA28 proteasome regulators in exacerbating the pathogenesis of DN by demonstrating that, compared with wild type/STZ mice, the Pa28*αβ*DKO/STZ mice do not develop severe albuminuria. Further testing provided that, compared to wild type, the OPN and MCP-1 inflammatory mediators are markedly suppressed in the Pa28*αβ*DKO/STZ glomeruli, mesangial cells, and RPC under high glucose conditions. OPN secretion was regulated by Pa28*α*/*β* and, among different vascular cell types, was only detectable in cultured RPC and mesangial cells exposed to high glucose. This is consistent with the higher sensitivity of RPC and mesangial cells to the cytotoxic effects of high glucose compared to other vascular cell types including the REC, KGEC, BPC, and BEC. These findings have strong implications in addressing why the trigger for the development of diabetic microvascular complications lies in the RPC and mesangial cells. The cell injury and death of these perivascular supporting cells in the retina and kidney caused by hyperglycemia precede the development of DR and DN. The increased OPN expression is reportedly associated with the development of DN and DR and pharmacological reagents capable of inhibiting OPN release provide protection against DN and DR [21, 42, 45]. OPN has macrophage chemoattractive function and the progression of DN and DR is facilitated by the recruitment of the macrophages and monocytes into retina and kidney. OPN release by perivascular cells in diabetic kidney and retina might regulate the local inflammatory cues to increase macrophage recruitment and activation. The novel function of PA28*α* and PA28*β* proteins in regulating OPN expression and diabetic microvascular injury demonstrates that, apart from their antigen processing properties, these proteins possess unexplored functions in sensing and responding to metabolic changes in a certain subset of vascular cells. Importantly, the increased expression of OPN following exposure to high glucose exclusively in RPC and mesangial cells that are main targets of diabetic hyperglycemia injury demonstrates a cell-specific function for PA28*α* and PA28*β* in sensing and responding to altered glucose levels.

Similar to LXR agonists, T0901317 and GW3965, that inhibit OPN expression and the severity of DN in STZ diabetic mice [42], the delivery of peptides that inhibit PA28 binding to 20S proteasomes, the XAPC7, HBX, and TAT-conjugated peptides inhibited OPN release from mesangial cells under high glucose conditions. This suggests that OPN expression under high glucose conditions from mesangial cells is regulated by PA28*α* and PA28*β* binding to the 20S proteasome. In agreement with the role of PA28*α* and PA28*β* in regulating the OPN expression, the reexpression of PA28*α* alone or PA28*α* and PA28*β* together in Pa28*αβ*DKO mesangial cells restored the OPN expression. Interestingly the expression of PA28*α* alone also stimulated the OPN release from the Pa28*αβ*DKO mesangial cells. This observation can be explained by the exceptional ability of the PA28*α* monomers to form an active regulatory complex capable of binding and activating the 20S proteasomes. Presently, it is unclear whether the reduced expression of OPN or MCP-1 alone is accountable for the ameliorated diabetic microvascular injury in Pa28*αβ*DKO mice or other pathways are also involved. Nevertheless, since other groups showed that proteasome inhibition in mice attenuates the development of DN [46–48], the relative inhibition of the proteasomes in Pa28*αβ*DKO RPC and mesangial cells would also provide a suitable explanation for their protection against diabetic renal and retinal injury. This can be supported by previous study in which the PA28 proteins were shown to be upregulated in RPC and also in diabetic glomeruli where expression level of PA28 proteins correlated with the severity of diabetic renal injury [1, 49]. It therefore appears that excessive activation or deregulation of the proteasomes by upregulated PA28 proteins could possibly result in microvascular damage in the retinal and glomerular vasculature.

Other investigators have shown that high glucose treatment of RPC and mesangial cells induces oxidative stress [50, 51]. The PA28*α* and PA28*β* were demonstrated to play key role in the clearance of misfolded and oxidized proteins and adaptation to oxidative stress in cultured cardiomyocytes [26]. Similarly, in RPC and mesangial cells, the PA28*α* and PA28*β* proteins could assist the RPC and mesangial cells in their adaptation to oxidative stress caused by high glucose. For instance, it was shown that RPC have lower proteasomal degradational capacity compared to other vascular cell types such as REC [1]. Therefore, PA28-*α*/-*β* proteins could assist the degradation of proteins damaged by exposure of RPC to high glucose but overt activation of the proteasomes in RPC and mesangial cells provokes tragic consequences.

## 4. Conclusions

This study provides major insights into the role of PA28*α* and PA28*β* genes in promoting diabetic renal and retinal microvascular injury. First of all, deletion of PA28*α* and PA28*β* genes protected diabetic animals against DN. Secondly, the expression of MCP-1 and OPN as important mediators of DN or DR was shown to be regulated by PA28*α* and PA28*β* in RPC and mesangial cells grown under high glucose. Thirdly, Pa28*αβ*DKO mice showed reduced severity of DR reflected by reduced number of acellular capillaries in their retinae. Lastly, the suppressive effect of PA28-inhibitory peptides on OPN release by mesangial cells grown in high glucose was demonstrated. Therefore, modulating the degradational activity of ubiquitin proteasome system mediated by PA28*α* and PA28*β* proteins offers a suitable candidate for the development of future treatments for microvascular complications of diabetes particularly in the retina and kidney.

## Figures and Tables

**Table 1:** Blood glucose levels in nondiabetic and STZ-injected diabetic mice.

Wild type		Pa28*αβ*DKO		
No STZ	4 m STZ	No STZ	4 m STZ	6 m STZ
139	473	135	483	527
177	569	123	467	483
164	452	226	504	505
192	527	179	513	539
184	568	146	476	543
150	492	152	535	569
182	480	139	432	476
129	493	182	528	481
164	559	165	542	509

The values represent the measured milligrams of glucose per deciliter of blood (mg/dL) in nine wild type and nine Pa28*αβ*DKO mice before and after four or six months of STZ injection.
